# Supplementary figures and images for: Changes in endolysosomal organization define a pre-degenerative state in the crumbs mutant Drosophila retina
Source: PLoS One. 2019 Dec 13;14(12):e0220220. doi: 10.1371/journal.pone.0220220 (PMC6910688; doi:10.1371/journal.pone.0220220)

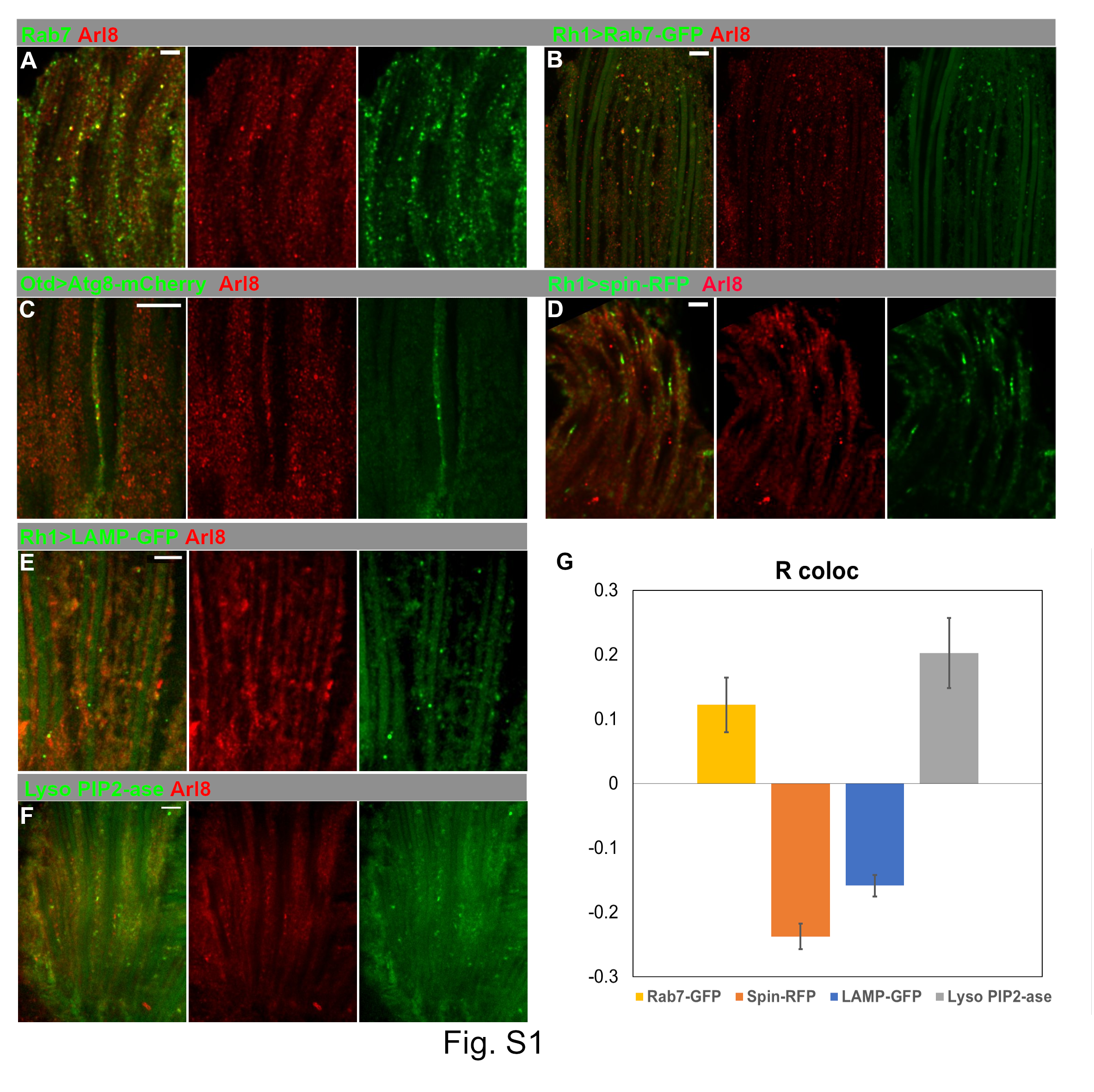

Supplement: S1 Fig — Longitudinal optical sections of w* retinas after 5 days of 12h light/12h dark conditions, stained with anti-Arl8 (red in all panels) and other lysosomal markers (green in all panels). (A, B) Arl8 occupies the same compartment as endogenous Rab7 (A) and Rh1-Gal4-mediated Rab7-GFP (B) in a sub-population of vesicles. R coloc for Rab7 and Arl8 (shown in Fig 5E) is ~0.17. (C) Arl8-positive compartments are often nearby, but non-identical to those labelled by Atg8-mCherry (green), an autophagosomal marker expressed in all photoreceptors via Otd-Gal4. R coloc for Atg8-mCherry and Arl8 (shown in Fig 6E) is ~0.05. (D) Arl8-positive compartments are often nearby, but non-identical with those labelled by the putative lysosomal transporter spinster (spin)-GFP (green), expressed by Rh1-Gal4 in photoreceptors R1-6. (E) Arl8-positive compartments are similarly adjacent, but largely non-identical to those labelled by the bona fide lysosomal marker, LAMP-GFP, expressed by Rh1-Gal4 in photoreceptors R1-6. (F) Arl8 overlaps with a subpopulation of vesicles carrying an intrinsic lysosomal marker, a GFP gene-trap insertion in the PIP2 phosphatase gene (Lyso PIP2-ase), CG6707. (G) Pearson’s colocalization coefficient (R coloc) between Arl8 and the markers shown, in retinas. For each stack, R coloc was calculated on regions (ROIs) of every 4th optical slice where clear vesicular staining was present, without obvious background from non-ommatidial tissue. The number of image stacks analysed for each comparison was as follows: Rab7-GFP n = 14 (270 regions); Spin-RFP n = 16 (614 regions); LAMP-GFP n = 13 (408 regions); LysoPIP2-ase n = 12 (233 regions). Scale bars: 5 μm. (TIF) [file pone.0220220.s001.tif]

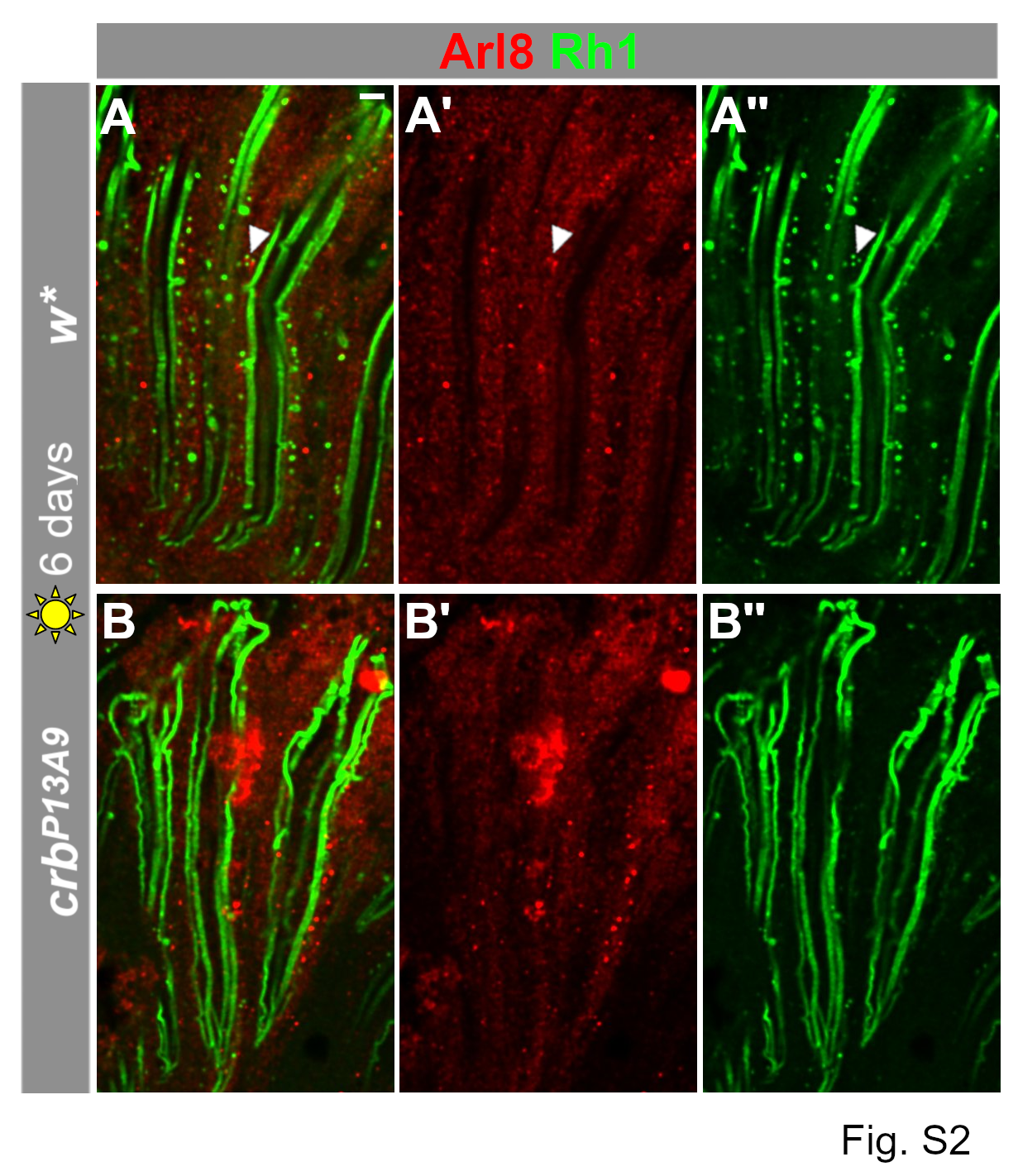

Supplement: S2 Fig — Longitudinal optical sections of w* and crbP13A9 retinas of flies kept for 6 days in constant light, stained for Arl8 (red) and Rh1 (green). Very few small Arl8-positive compartments are also Rh1-positive (A-A”; arrowhead), as is the case in normal light conditions (see Fig 4). In contrast, large Arl8-positive patches in crbP13A9 are always negative for Rh1 (B). (TIF) [file pone.0220220.s002.tif]

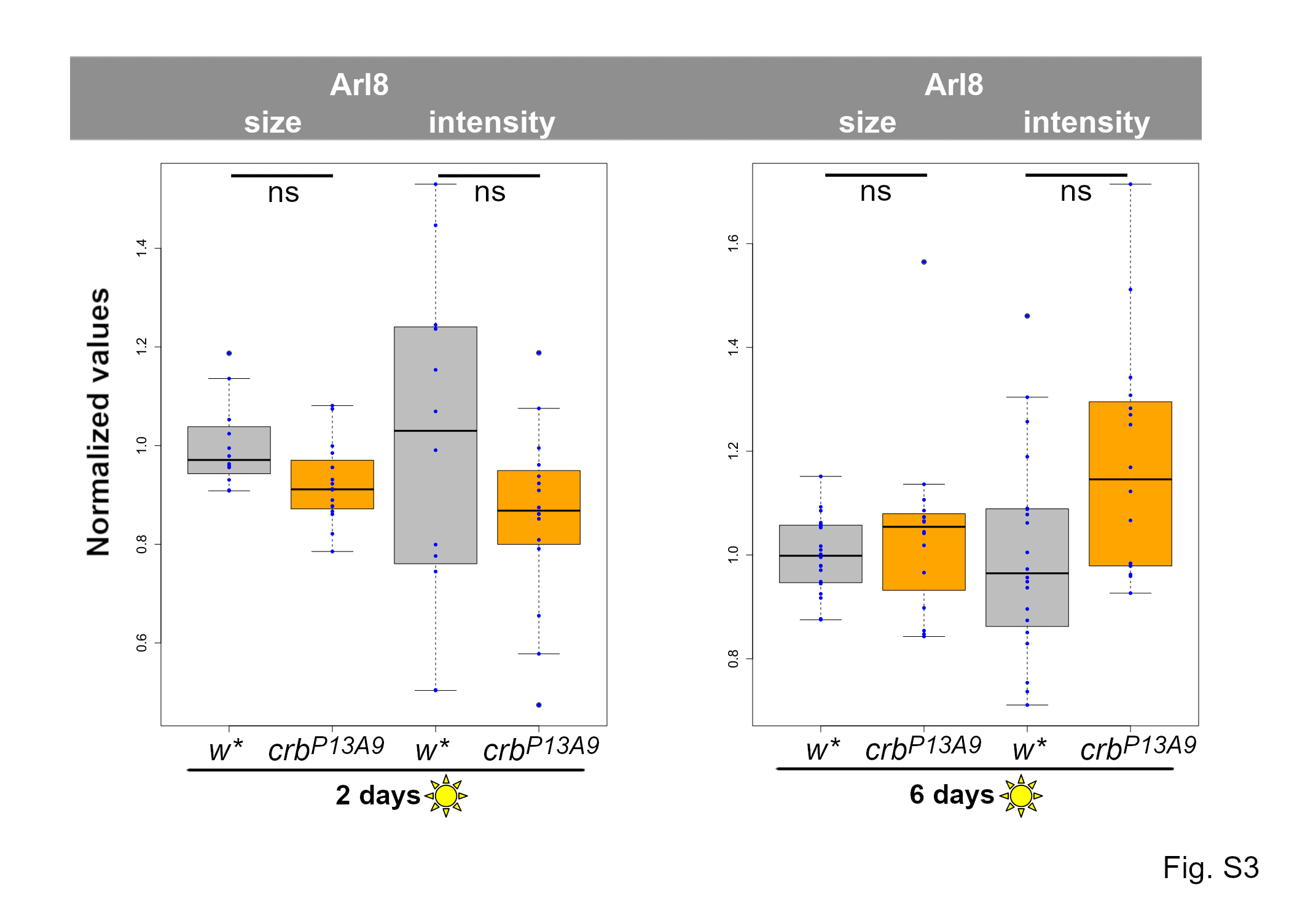

Supplement: S3 Fig — Arl8 compartments, quantified as in Figs 2 and 3, show no significant differences in size and fluorescence intensity between w* (grey box plots) and crbP13A9 (orange box plots) after two (left graph) or six days (right graph) of constant intense light stress. “ns” indicates not significant (P>0.01). For 2 days constant light, w* n = 12 image stacks and crbP13A9 n = 16 image stacks; for 6 days constant light, w* n = 20 image stacks and crbP13A9 n = 16 image stacks. (TIF) [file pone.0220220.s003.tif]
